# Microstructural and Hydrophilic Properties of Polylactide Polymer Samples with Various 3D Printing Patterns

**DOI:** 10.3390/polym16091281

**Published:** 2024-05-03

**Authors:** Alexandr S. Lenshin, Vera E. Frolova, Sergey V. Kannykin, Evelina P. Domashevskaya

**Affiliations:** Department of Solid State Physics and Nanostructures, Voronezh State University, Voronezh 394018, Russia; lenshinas@mail.ru (A.S.L.); ternovaya@phys.vsu.ru (V.E.F.); svkannykin@gmail.com (S.V.K.)

**Keywords:** polylactide (polylactic acid-PLA), 3D printing model drawings, X-ray phase state, partial crystallization, stable phase of ᾳ-Poly(L-lactide), IR spectra, surface wetting

## Abstract

The aim of the work is to study the effect of the 3D printing process on the microstructural and hydrophilic properties of polylactic acid (PLA) samples with various model printing patterns obtained from the black filament PLA by sequentially applying polymer layers using the FDM (fused deposition modeling) method. X-ray phase analysis revealed the partial crystallization of PLA polymer chains in the printed samples, which occurs under thermal and mechanical action on the original amorphous PLA filament during 3D printing to varying degrees, depending on the geometry of the pattern and the morphology of its surface. At the same time, IR spectroscopy data indicate the preservation of all intrastructural chemical bonds of polylactide. Measured at the original installation, the values of the wetting edge angles on the surface of the printed samples are in the range φ = 50–60°, which is significantly less than the right angle. This indicates the hydrophilic properties of the whole sample’s surface. At the same time, the influence of different geometries of model drawings in printed samples was found not only on the morphology of the sample’s surface according to SEM data but also on its wettability.

## 1. Introduction

Recently, polylactide (polylactic acid, PLA) has attracted increasing attention form researchers since it is one of the most promising biodegradable thermoplastic polymers [1,2,3,4]. Polylactide is a thermoplastic, aliphatic polyester, the monomer of which is lactic acid. Recent publications present the results of studies of the synthesis, as well as the physicochemical and mechanical properties, of PLA using the thermography method, X-ray diffraction analysis, IR and UV spectroscopy, nuclear magnetic resonance NMR, and many other methods [1,2,3,4].

Polylactide was invented in the 1930s, but its commercial production began fifty years later. Recently, the use of this material as both a packaging material and a starting material—i.e., a filament for 3D printing—has grown to a huge scale. In popularity, it has become the number one bioplastic in the world. The versatility of PLA is closely related to one of its main advantages, namely, its ability to biodegrade.

In the form of a filament, PLA is widely used in 3D printing; it is easy to handle, so it is most suitable for novice operators.

PLA has relatively low thermal and mechanical resistance. In addition, it is hygroscopic, which can make a PLA product potentially fragile, and if used incorrectly, it can cause some problems, such as the clogging of the extruder nozzle. Nevertheless, when using PLA, the 3D printing process is accelerated due to relatively lower printing temperatures, i.e., from 180 to 230 °C compared to other thermoplastics [3,4].

In addition, PLA is intensively used in food and industrial packaging [3], and it can successfully replace traditional petroleum-based plastics such as polystyrene, polyethylene, polypropylene, and polyethylene terephthalate in many single-use or short-term applications [4]. The advantage of PLA over other thermoplastic materials is its biological origin from polylactic acid. Therefore, PLA is made entirely from renewable agricultural products and belongs to the class of biopolymers capable of biodegradation.

PLA exhibits good mechanical properties such as high strength, high Young’s modulus, and high biocompatibility [5].

The PLA polymer is made from organic renewable raw materials—soy protein, corn, and cane—making production inexpensive. By adjusting the lactic acid level during the production of polylactide, it is possible to identify various properties of the polymer, thus expanding the scope of its use. The study of the various properties of PLA shows that it can be an effective polymer for use in various fields: biomedicine, packaging, electrical engineering, etc. Of all these applications, the biomedical ones have been widely studied, while the study of the other applications is still at an early stage. Being a biodegradable and biocompatible polymer, it may not cause environmental pollution [6].

Natural PLA is an opaque plastic of a cloudy light shade, with a glass transition temperature of 60 °C, resistance to temperatures up to 70 °C, and high mechanical strength, flexibility, and elasticity. Both lactic acid and polylactide exhibit optical activity; thus, they exist in the form of two L- and D-stereoisomers, which are mirror images of each other. By varying the relative content of these forms in polylactide, it is possible to set the properties of the resulting polymer to obtain various classes of polylactide materials. Polylactide made of 100% L-lactide (L-PLA) has a high degree of stereoregularity, which gives it a crystallinity with a density of 1290 g/cm^3^. Using a mixture of D- and L-forms of lactide during polymerization, an amorphous polylactide (L,D-PLA) with a density of 1.248 g/cm^3^ can be obtained [7].

The polylactide of 100% L-lactide (L-PLA) has a high degree of stereoregularity, which provides the crystalline form of an orthorhombic-phase α-Poly(L-lactide) with the chemical formula (C_3_H_5_O_3_)_n_ and the following unit cell parameters: a = 10.61, b = 6.05, and c = 28.8 Ǻ (PDF-2-2006 card number 00-054-1917, Diffract Plus 2005). Thus, it follows from the literature review [1] that the α-phase is more stable as compared to another known phase of the β-Poly(L-Lactide) polylactide, also with an orthorhombic unit cell, but with other unit cell parameters, i.e., a = 10.3, b = 18.2, c = 9.0 Ǻ.

The most important advantage of using PLA is safety; it is completely eco-friendly, biocompatible, and biodegradable for about six months under the right conditions; there-fore, it is safe for the use in medicine and the food industry and can also be recycled and reused [8].

Because PLA is a biodegradable plastic, it can be degraded over time by microorganisms similar to those used in industrial composting. As a result of this organic process, PLA is broken down into smaller harmless molecules such as carbon dioxide and water. The time during which this process takes place, measured over several months, strongly depends on both environmental conditions and the material itself. Another way of processing PLA is associated with the distinctive feature of thermoplastics used in 3D printing, melting, and re-extruding without significant loss of material, such as, for example, in the works [9,10].

In addition, PLA belongs to polar polymers, the links of which have significant polarity due to the presence of OH groups in the structural formula [1]. Polarity is the ratio of positive and negative charges. It defines the mechanisms of interaction of the polymer with the environment, and in particular with water. Polar polymers are hydrophilic as a result of the formation of hydrogen bonds with water molecules, while non-polar polymers are hydrophobic; that is, they repel water [11,12]. For example, the high hydrophilicity of cellulose (C_6_H_10_O_5_)_n_ is due to the presence of three OH groups in the elementary unit of macromolecules, which interact with water molecules by forming hydrogen bonds [11]. These properties of the interaction of polymers with water are not only fundamentally important, they can also be widely used in industry to produce products or coatings with the desired effect.

Polylactide for 3D printing is produced by most manufacturers of consumables for personal 3D printers in the form of a filament PLA in a wide range of colors. At the same time, unpainted PLA has the lowest degree of crystallinity and the highest mechanical strength compared to the painted versions [13].

However, any 3D printer, even the most entry-level, is capable of working with these materials.

The aim of this work is to study the effect of the 3D printing process on the microstructural and hydrophilic properties of PLA samples with various model printing patterns obtained from black filament PLA by sequentially applying polymer layers using the FDM (fused deposition modeling) method.

## 2. Materials and Methods

### 2.1. Materials and 3D Printing Conditions

We produced the samples for the study using the most common 3D printing technology, which uses fused filament fabrication (FFF) molten thermoplastic filament as a raw material.

**Figure 1:**
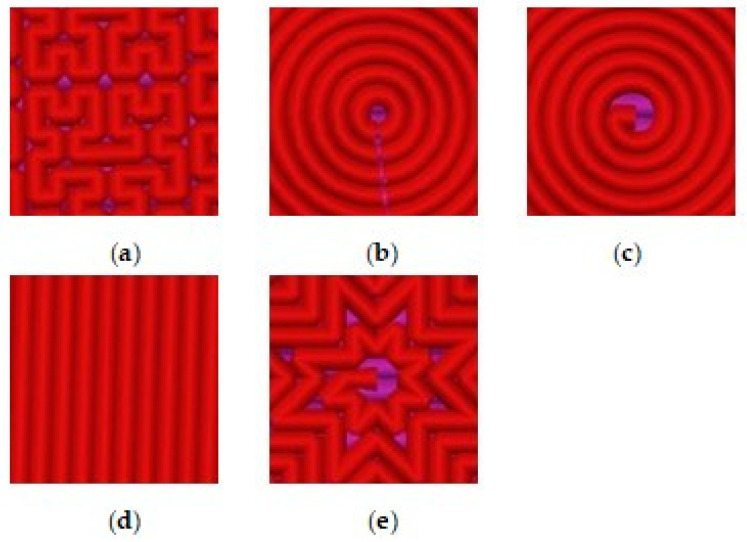
Five types of model patterns used in 3D printing of samples from filament PLA: (**a**) 1_Hilbert; (**b**) 2_Concentric; (**c**) 3_Archimedean; (**d**) 4_Rectilinear; (**e**) 5_Octagram.

The samples were made from black polylactide filament (filament PLA) with a diameter of 1.75 mm on a Hercules Original 3D printer(IMPRINTA, Moscow, Russia) via layered FDM (fused deposition modeling)polymer layers [1,2,3,4].The temperature of the extruder is 230 °C, the power is 500 W, the nozzle diameter is 0.5 mm, and the printing speed is of 40 mm/sec. The thickness of the printed cylindrical samples is 5 mm, the diameter of the samples is 20 mm. The thickness of the initial layer is 0.35 mm, and the thickness of the subsequent layers is 0.30 mm. The percentage of sample filling is 20%. Before starting 3D printing of samples, we heated the table for printing up to the 95 °C. Five cylindrical samples with a diameter of 20 mm and a thickness of 5 mm were printed. The printed samples were distinguished by 3D printing model patterns. Figure 1 shows five types of the model patterns used in 3D printing of the samples from PLA filament.

Along with the printed samples, the initial sample of the filament PLA was studied. The results of the study of the microstructural properties for six samples will be presented in the next section.

### 2.2. Methods

Scanning electron microscopy (SEM) of the surfaces of five printed samples with different model patterns was performed with a scanning electron microscope JSM 6510LV (JEOL, Tokyo, Japan).

To obtain a better contrast on SEM microphotographs, studies of the morphology of the surfaces of the printed samples were carried out for the surfaces of samples coated with the thinnest layer of gold, several nanometers thick, with different magnifications:×40, ×500, ×1000.

The phase composition and structure of the printed samples and the initial filament PLA was analyzed via an X-ray diffraction (XRD) method on an ARL X’TRA, (Thermo Fisher Scientific, Basel, Switzerland) with a monochromator operating in parallel beam geometry and in the mode θ−θ within the angle range of 2θ = 2–40°, Cu Kα_1_ radiation at high voltage (U = 29 kV), and the anode current of the X-ray tube (I = 25 mA).

Infrared (IR) spectroscopy is a non-destructive optical method used to solve specific problems, including determining the fundamental characteristics of a molecule, quantitative analysis of the known phases in a substance, identification of chemical compounds, and elucidation of their structure. This optical method is based on measuring the intensity of infrared radiation absorbed or reflected by a certain material, which is associated with vibrational and rotational vibrations of molecular fragments and manifests itself in the intensity distribution in the absorption bands depending on the wavelength (λ) or its inverse value, which is known as the wave number (v).

Studies of the molecular structure were carried out for five printed samples with different model patterns and the initial filament PLA by measuring the transmission infrared (TIR) spectra by the attenuated total internal reflection method with the IR Fourier spectrometer Brucker Vertex 70 (Bruker Optics, Ettlingen, Germany) in the range of 4000–400 cm^−1^.

**Figure 2:**
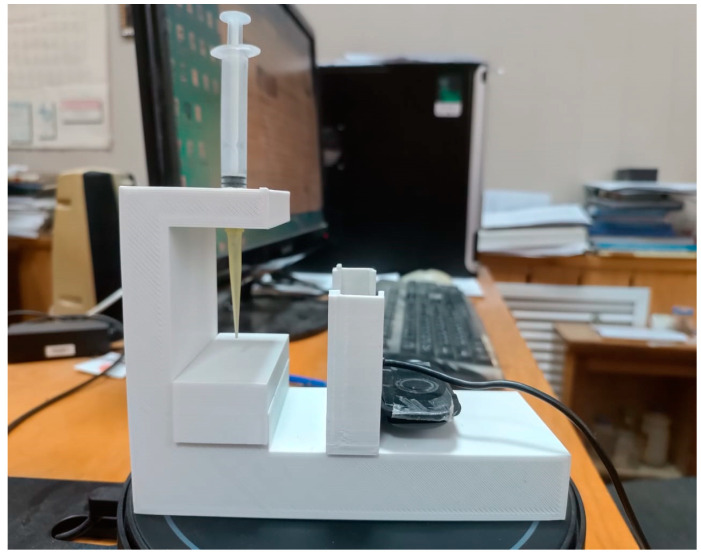
General view of the original installation for measuring the wetting edge angles.

The wettability of the surfaces of flat printed samples with various 3D printing patterns was studied using the original installation for measuring the edge angles of wetting (Figure 2), which we manufactured with a 3D printer. The installation is a stand with a sample holder on which a flat sample is placed. A drop gauge is installed on top, which creates droplets on the surfaces of the samples to measure the wetting edge angle. A webcam is installed opposite the stand with the sample under study, which displays an image of a drop on the screen, and using the PicPick graphic editor program, the wetting edge angle φ of the sample is measured.

**Figure 3:**
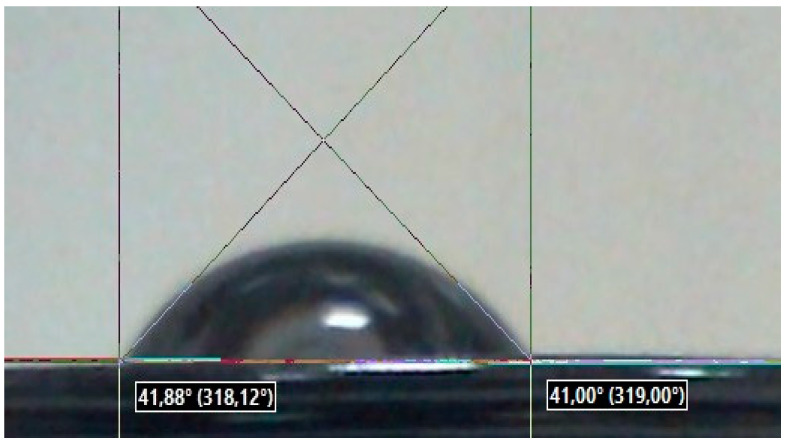
The wetting edge angle φ on the hydrophilic surface.

A drop of liquid on the surface of a solid, depending on the nature of the sample, the liquid, and the medium where it is located, can spread completely or partially and acquire such a form as shown in Figure 3. The angle φ between the tangent of the surface of the drop and the surface of a solid body, measured towards the surface of the drop, is called the wetting edge angle φ [13,14].

If a drop of liquid completely or partially spreads over the surface of the sample and forms an acute angle φ < 90° with it, as shown in Figure 3, then the liquid moistens this surface. Only those liquids that reduce the surface tension of a given solid at the boundary with air moisten the solid surface. Surfaces of solids wetted with water are called hydrophilic. Surfaces over which the liquid does not spread and the drop forms with it an obtuse edge angle φ > 90° are called hydrophobic.

The accuracy of measuring the wetting edge angles φ in our original installation was about 1 degree.

If a liquid drop completely or partially spreads over the sample surface and forms an acute angle θ < 90° with it, as is shown in Figure 3, then the liquid wets this surface. Only those liquids can moisten a solid surface that has a lower surface tension than a given solid at the boundary with air. Surfaces of solids wetted by water are called hydrophilic.

Surfaces over which water does not spread and instead forms an obtuse contact angle θ > 90° are called hydrophobic.

## 3. Results and Discussion

### 3.1. Scanning Electron Microscopy

**Figure 4:**
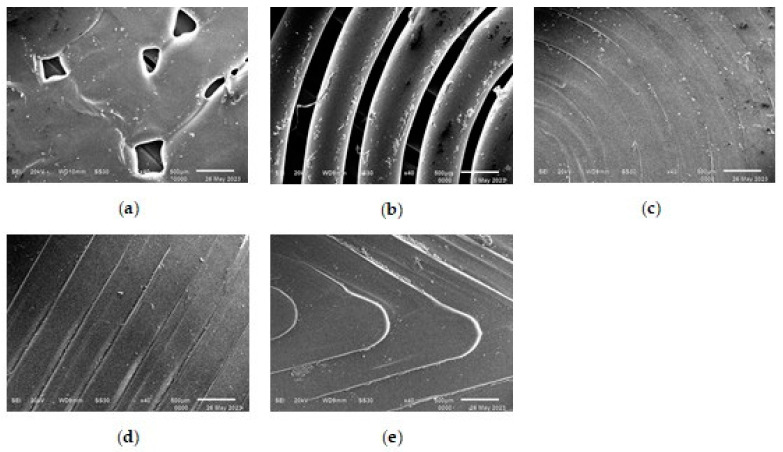
SEM micrographs with ×40 magnification for samples printed with various model drawings. Wetting edge angle φ on the hydrophilic surface: (**a**) 1_Hilbert; (**b**) 2_Concentric; (**c**) 3_Archimedean; (**d**) 4_Rectilinear; (**e**) 5_Octagram.

Figure 4 shows SEM micrographs obtained with a magnification of ×40 for five samples printed from filament PLA with different model patterns.

The results of the SEM show that the micrograph of the 1_Hilbert sample surface least represents the most complex geometry of its model pattern. At the smeared surface of this sample, the results show a large number of microdefects of triangular and rectangular shapes that occur at complex angular turns of the model pattern when scanning the extruder. And the greatest contrast comes with the micrograph of the sample with the 2_Concentric pattern, representing the clear relief of the concentric circles of PLA with deep dips between them of a smaller width.

The other three micrographs of the surfaces of the samples 3_Archimedean, 4_Rectilinear, and 5_Octagram represent the geometry of their model drawings more or less equally.

Thus, a question arises as to whether the geometry of the model pattern, manifested in the morphology of the surface, affects the atomic structure of the printed samples and the wettability of their surfaces. The answer to this question is contained in the following sections of our work.

### 3.2. XRD Phase and Structure Analysis

**Figure 5:**
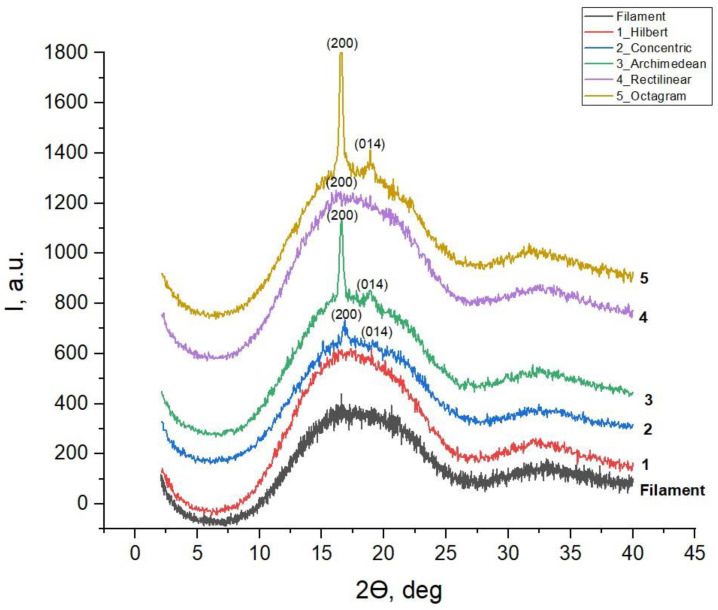
Diffractograms of the original filament PLA and printed samples with various 3D printing model patterns.

Figure 5 shows the results of a study of the crystal state in the printed samples with five different 3D printing patterns and the initial filament PLAobtained via X-ray diffraction (XRD) in the range of Bragg angles 2θ = 2–40°. 

The obtained results show that the diffractograms of all printed samples, as well as the original filament PLA, include two wide reflection bands (halos) from the amorphous phase of PLA, with the most-intense band in the range of angles 2θ ≈ 10–25°, and the second, less-intense band in the range of 2θ ≈ 30–40° (Figure 5). Against the background of these wide bands, three out of five printed samples have one or two narrow diffraction lines from the crystalline phase of α-Poly(L-lactide) with the chemical formula (C_3_H_5_O_3_)_n_ and an orthorhombic structure (according to ICDD Card: 00-054-1917 [15]), with the following unit cell parameters: a = 10.61, b = 6.05 and c = 28.8 Ǻ. It follows from the literature review [1] that this phase is more stable compared to another known phase of β-Poly(D,L-Lactide), also with an orthorhombic unit cell, but with other unit cell parameters: a = 10.3, b = 18.2, c = 9.0 Ǻ.

Table 1 shows the values of the Bragg angles 2θ and the interplane distances d (Ǻ) of the two most-intense lines of the orthorhombic phase of the α-Poly(L-lactide), which appear in some printed samples. The last column of Table 1 contains literature data on the interplanar distances and indices of these two lines from the international ICDD Card database [15].

The results presented in Table 1 and Figure 5 show that the sample with the 1_Hilbert pattern and the most-blurred surface morphology (Figure 4) remains amorphous, as does the original filament PLA. In the sample with the 4_Rectilinear pattern, the most-intense diffraction line (200) of the crystalline α-phase, with an interplane distance of d = 5.30851 Ǻ, is barely outlined, and in the sample with the most contrasting 2_Concentric pattern, it becomes the only noticeable line (these questionable lines are marked with a “?” sign in Table 1). But the highest relative intensity against the background of an amorphous halo is shown by this line (200) in samples with figures 3_Archimedean and 5_Octagram, along with the appearance of the second intense line (014) of this crystalline α-phase [15].

Two noticeable X-ray lines (200) and (014) of approximately the same intensity from the crystalline phase of α-Poly(L-lactide) against the background of a significantly superior peak from the amorphous phase of PLA show those two samples with model patterns 3_Archimedean and 5_Octagram, in which the extruder continuously layers from the center of the sample to its edge. This continuous, layered drawing process contributes to the alignment of some of the filaments in the preferred direction and the partial crystallization of PLA in such printed samples.

Previously, in [12], it was shown that the level of atomic organization in PLA strongly depends on the conditions of sample preparation and processing. Thus, the volumetric PLA used by the authors as a reference involved, along with the amorphous polycrystalline c phase α-Poly(L-lactide),the diffractogram, of which seven diffraction lines—with the two most intense lines (200) at 2θ = 16.8° and (014) at 2θ =19.2°—were registered against the background of an amorphous halo. These two lines with indices (200) and (014) are in good agreement with our data shown in Table 1 and Figure 5.

At the same time, the structure of the sample with misoriented nanofibers obtained via electro scission in another work [16] turned out to be amorphous, with two typical wide X-ray bands (halos) in the regions 2θ ≈ 10–25° and 2θ ≈ 30–40°, coinciding with our halos in Figure 5, since the rapid hardening of the fibers in the process of electro screening limited the long-term three-dimensional order at the atomic level of PLA [16].

However, after annealing the nanofibers below the melting point of PLA, a line (200) appeared on the diffractogram of the annealed sample at 2θ = 16.8° from the crystalline phase of ᾳ-Poly(L-lactide), which, according to the authors [16], indicated self-assembly of PLA polymer chains at the atomic level.

Similar differences found by us in the diffractograms of the printed samples from the diffractogram of the original filament PLA are due to the partial crystallization of the initially misoriented polymer chains of amorphous filament PLA, which occurs in the extruder under thermal and mechanical effects on the original filamentous sample during 3D printing.

At the same time, the most noticeable ordering under the appearance of the orthorhombic phase of α-Poly(L-lactide) occurs in the printed samples with precisely those drawings—3_Archimedean and 5_Octagram—where the extruder operates continuously within each layer, with the layered set of the full thickness of the samples being equal to 5 mm. This is how the influence of the 3D printing model pattern affects the atomic structure and the phase composition of the PLA polylactide, the initial filament of which is amorphous PLA.

Here, we should compare the results obtained from the X-ray phase state of the PLA samples with different model patterns with the corresponding results for the samples with the same 3D printing patterns printed from a similar polymer polyethylene terephthalate glycol (PETG), with traces of polycrystalline coloring white-pigment rutile TiO_2_, which we obtained in our previous work [17].

Unlike the results presented in this paper on the varying degrees of influence of the 3D printing model pattern on the X-ray phase state of the PLA in the printed samples in the form of more- or less-noticeable crystallization against the background of the main amorphous phase, the effect of the type of model pattern on the amorphous state of PETG was not detected. Thermal and mechanical effects on the original PETG filament during the 3D printing of the samples with the same different model patterns as those of the PLA samples were manifested in the same way for all five drawings. This consisted of an increase in the relative intensity of the main diffraction maximum (halo) of amorphous PETG by an order of magnitude in the printed samples compared to the original amorphous filament, which was due to the greater ordering of the polymer chains of amorphous PETG in the printed samples. No traces of crystallization of the samples during 3D printing from the amorphous PETG filament were found [18].

### 3.3. IR Spectroscopy

IR spectroscopy is a universal method for obtaining information about the molecular structure of substances, and it allows us to determine the nature of atomic grouping, the nature of chemical bonds, and their changes under the influence of external conditions [18,19]. Any molecule has its own individual oscillation spectrum; therefore, by comparing the modes of the obtained experimental spectrum with known literature data, it is possible to identify the substance under study.

**Figure 6:**
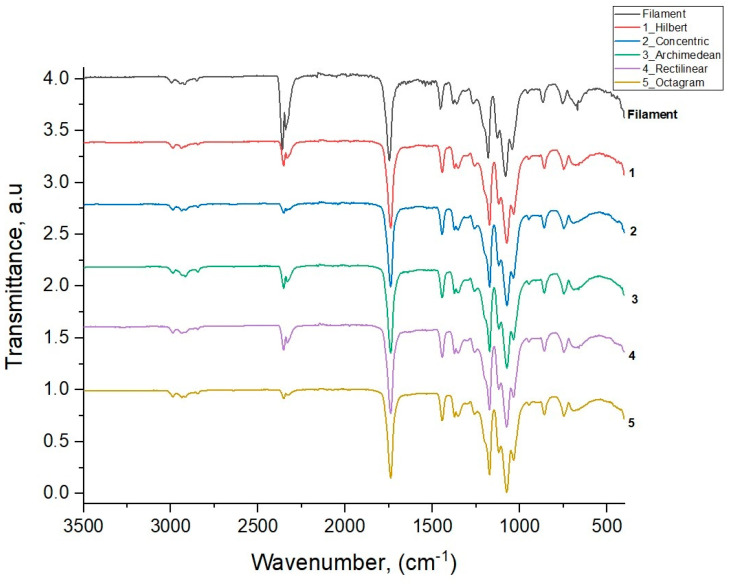
IR transmission spectra for the original filament PLA and five printed samples with different 3D printing model patterns.

Figure 6 shows the IR transmission spectra for the initial filament PLA and five 3D-printed samples with various model patterns obtained through the method of attenuated total internal reflection [20,21] with the IR spectrometer Brucker Vertex 70.

Table 2 shows the results of the assignment for all the vibration modes of the studied samples, along with the literature data for PLA polymer modes from [22], given in the last column.

The results of IR spectroscopy show that the wave numbers and relative intensities of the vibration modes of all five printed samples with different patterns have similar values and correspond to the values of the main modes of the original PLA filament used in the 3D printing of the samples and the literature data for PLA polymer modes [22]. This means that the intrastructural chemical bonds of the PLA polymer are not subjected to mechanical and thermal effects during the 3D printing process. These effects affect only the degree of ordering of the polymer chains and manifest themselves in the partial crystallization of the amorphous polymer PLA, which is more or less noticeable in the printed samples with different model patterns compared to the original amorphous filament PLA sample.

### 3.4. Wettability of the Printed PLA Samples’ Surfaces with Various Model Patterns

**Figure 7:**
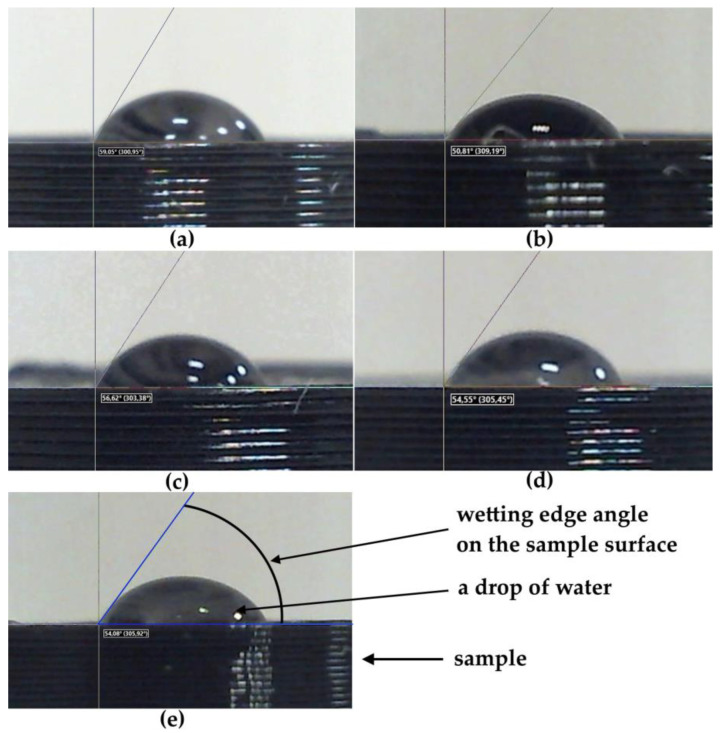
Images of droplets in the process of determining the wetting edge angle on the surfaces of five samples printed from a PLA filament with various model patterns: (**a**) 1_Hilbert; (**b**) 2_Concentric, (**c**) 3_Archimedean; (**d**) 4_Rectilinear;(**e**) 5_Octagram.

The wettability of the sample surface is a manifestation of the intermolecular interaction at the interface of three phases—solid, liquid, and gas—expressed in the spreading of liquid on the surface of a solid. Since the measurement of the wetting edge angle of the surface is carried out only on flat samples, this section presents the results of a wettability study for only five printed samples. Figure 7 shows images of droplets on the screen of the installation while measuring the wetting edge angle on the surfaces of five samples with different 3D printing patterns made of PLA polymer.

Measurements of the wetting edge angles for water droplets on the surfaces of the samples were carried out at five points foreach sample, with an accuracy of one degree. In Table 3, we shown the average values of these angles (φ).

A comparative analysis of the values of the wetting edge angles for the surfaces of samples with different model patterns shows that the wetting edge angles of all printed samples vary within φ = 50°–60°. The large deviation of the average wetting angle values for all samples with respect to the right angle of 90° shows that the surfaces of all five printed samples with different patterns are wettable, i.e., hydrophilic. At the same time, three samples with figures 3_Archimedean, 4_Rectilinear, and 5_Octagram, whose surface micrographs in Figure 4 represent the geometry of their model drawings more or less adequately, have almost equal values of the wetting edge angles φ ≈ 55°. The sample with the least-pronounced 1_Hilbert pattern on a smoother surface with individual defects shows the highest value of the wetting angle φ = 59°; i.e., it is less hydrophilic as compared to the other samples. The 1_Hilbert model pattern has the most complex geometry, which the extruder cannot implement in a continuously layered way. Therefore, when printing a sample with such a pattern, there is a statistically noticeable destruction of the polar bonds of PLA and the resulting decrease in hydrophilicity.

While the sample with the most contrasting pattern of concentric circles on the surface, separated by noticeable concentric dips, 2_Concentric, shows the lowest value of the wetting angle φ = 50°, in comparison to the samples with other patterns, it is most hydrophilic.

Thus, a positive qualitative answer was obtained to the question of whether the geometry of the model pattern, manifested in the morphology of the sample surface, affects the wettability of its surface. It should be recalled here that in our previous work [13], we obtained similar but almost identical values of the edge angles of surface wetting for samples with the same model patterns (φ ≈ 50°) but did not find a noticeable effect of the geometry of the model pattern on the wettability of the surface of samples printed from PETG polymer.

And since the wettability of the solid surface is a manifestation of intermolecular interactions at the interface of liquid contact with the solid surface, it could be assumed that one of the mechanisms of such an interaction may be the participation of hydrogen groups of the more-plastic polylactide PLA in the formation of more- or less-strong hydrogen bonds with water molecules on the surfaces of all five samples, leading to a significant decrease in the marginal the wetting angles relative to 90° and the hydrophilicity of the printed samples.

## 4. Conclusions

The results of the study for the printed samples obtained from the PLA filament by sequentially applying layers of polylactic acid polymer with various model patterns using the fused deposition modeling method on a Hercules Original 3D printer at the extruder temperature of 230 °C and a power of 500 W revealed a different degree of influence of the 3D printing model pattern on their microstructural and hydrophilic properties using methods of X-ray diffraction, IR spectroscopy, and measurement of the wetting edge angle.

The differences we found between the phase composites of the printed samples and the original filament PLA are due to the partial crystallization of the initially misoriented polymer chains of amorphous PLA, with the appearance, in some samples, of the orthorhombic phase ᾳ-Poly(L-lactide), which occurs under thermal and mechanical influences on the original amorphous filament PLA during 3D printing.

The most noticeable crystallization with the appearance of orthorhombic ᾳ-phase of polylactide occurs in the printed samples with those 3_Archimedean and 5_Octagram patterns, which the extruder performs continuously within each layer with a continuously layered set of full-thickness samples (5 mm). In this way, the influence of the 3D printing model pattern on the atomic structure and phase composition of the initial amorphous filament PLA is manifested.

Regardless of the partial crystallization and corresponding changes in the phase composition of some printed samples, the intrastructural chemical bonds of PLA polylactide are not subjected to noticeable effects of the 3D printing process. Therefore, the wave numbers and relative intensities of the IR oscillation modes of all five printed samples with different model patterns have almost identical values and good agreement with the corresponding values of the main modes for the original filament PLA and the literature data.

The values of the edge angles of wetting water droplets on the surfaces of all printed samples measured at the original installation are within φ = 50°–60°, significantly less than the right angle θ = 90°, which corresponds to the hydrophilic properties of the surfaces of the printed samples.

With the general hydrophilicity of all the printed samples, the influence of different geometries of the model pattern was found to have an effect not only on the morphology of the surface—according to SEM micrographs—but also on its wettability. Three samples with figures 3_Archimedean, 4_Rectilinear, and 5_Octagram, whose surfaces represent the geometry of their model drawings more or less adequately, have almost equal values of the wetting edge angles φ ≈ 55°.

The 1_Hilbert model pattern has the most complex geometry, which the extruder cannot implement in a continuously layered way. Therefore, when printing a sample with such a pattern, there is a statistically noticeable destruction of the polar bonds of the PLA, with the resulting decrease in hydrophilicity, and the sample with this pattern shows the highest value of the wetting angle φ = 59°; i.e., it is less hydrophilic compared to the others. The sample with the simplest and contrasting 2_Concentric pattern shows the lowest value of the wetting angle φ = 50°, and in relation to samples with other patterns, it is the most hydrophilic.

One of the mechanisms of intermolecular interaction at the boundary of contact of the water droplet with the surfaces of the printed samples may be the participation of PLA hydrogen groups in the formation of more- or less-strong hydrogen bonds with water molecules on the surfaces of samples, leading to a significant decrease in the wetting edge angles relative to 90° and the hydrophilicity of the printed samples.

Thus, we have shown that the 3D printing process leads to the orientation of polylactide polymer chains with more- or less-noticeable partial crystallization in some samples, depending on the geometry of the model pattern, as a result of the statistically significant alignment of PLA molecules caused by extrusion at a temperature of 230 °C. At the same time, the intrastructural chemical bonds of PLA are preserved in all the study’s printed samples with different model patterns, as in the original filament.

## Figures and Tables

**Table 1:** Values of Bragg angles 2θ and interplane distances d (Ǻ) of the two most-intense lines of the orthorhombic crystalline phase of the PLA α-Poly(L-lactide) in the printed samples with various model patterns.

PLA Sample with the Modeled Image	2θ, (°)	d (Ǻ)	d (Ǻ) ICDD Card: 00-054-1917 [15]
1_Hilbert	-	-	-
	-	-	-
2_Concentric	16, 75	5, 29	5, 30851 (200)
	18, 85 ?	4, 70 ?	4, 64173 (014)
3_Archimedean	16, 55	5, 35	5, 30851 (200)
	18, 80	4, 70	4, 64173 (014)
4_Rectilinear	16, 75 ?	5, 31 ?	5, 30851 (200)
	-	-	
5_Octagram	16, 60	5, 33	5, 30851 (200)
	18, 80	4, 71	4, 64173 (014)
Fiber (filament)	-	-	-
	-	-	-

**Table 2:** Identification of the IR spectra vibration modes of the initial filament PLA and printed samples with different model patterns.

Identification of Vibration Modes	Vibration Modes of the Original Filament PLA and Printed Sample Samples, cm^−1^						
	Filament PLA	1_Hilbert	2_Concentric	3_Archimedian	4_Rectilinear	5_Octagram	PLA [22]
Valence vibrations of C=O bonds in the carbonyl group C=O	667	688	692	690	682	690	
	695	757	753	752	752	750	752
Symmetric valence vibrations of aliphatic and aromatic C-H bond	865	864	867	865	869	865	
C-CH_3_ helical mainline vibrations	954	954	954	952	956	954	
	1043	1039	1045	1043	1045	1045	1045
The methylene group and vibrations of the C-O-C ester bond	1083	1080	1081	1080	1082	1082	1090
Valence vibrations of C=O bonds in the carbonyl group	1126	1124	1126	1130	1126	1126	1130
	1267	1261	1267	1265	1263	1270	1265
Symmetric CH_3_ bonds	1359	1355	1360	1361	1359	1357	1360
	2995	3003	2999	2993	2999	2993	2997
Symmetric valence vibrations of aliphatic and aromatic C-H bond	1379	1384	1379	1380	1382	1382	
Asymmetric CH_3_ bonds	1456	1456	1452	1455	1452	1450	1452
	2947	2939	2945	2923	2939	2931	2947
Valence vibrations of carbonyl C=O bond	1747	1745	1747	1747	1747	147	1747
Symmetrical stretching of the CH bond	2854	2848	2852	2850	2852	2850	2882
Asymmetric CH_3_ bond	2947	2939	2945	2923	2939	2931	2947

**Table 3:** Average values of the wetting angles of water droplets on the surfaces of printed polylactide (PLA) samples with different 3D printing patterns.

Model Drawing	The Average Value of the Wetting Edge Angle φ, Degree
1_Hilbert	59
2_Concentric	50
3_Archimedean	56
4_Rectilinear	55
5_Octagram	55

## Data Availability

Data are contained within the article.
